# Analysis and Modeling for Big Data in Cancer Research

**DOI:** 10.1155/2017/1972097

**Published:** 2017-06-12

**Authors:** Bing Niu, Peter B. Harrington, Guozheng Li, Jianxin Li, Simon Poon

**Affiliations:** ^1^College of Life Science, Shanghai University, Shanghai, China; ^2^Center for Intelligent Chemical Instrumentation, Department of Chemistry and Biochemistry, Ohio University, Athens, OH, USA; ^3^China Academy of Chinese Medical Sciences, Beijing, China; ^4^Big Data Research Group, School of Computer Science & Software Engineering, The University of Western Australia (Go8), Perth, WA, Australia; ^5^School of Information Technologies, University of Sydney, Sydney, NSW, Australia

Cancer is a major disease which has become the biggest threat to human health due to its difficult early detection, diagnosis, and treatment. According to the survey of the World Health Organization in 2012, there were four million new cancer cases and 8.2 million cancer-related deaths worldwide. The history of treatment of tumors covered traditional herbal medicines, surgical anatomy, antitumor chemotherapy/radiotherapy, and new targeted drug therapy and immunotherapy. In the past few decades, with the rapid development of high-throughput technologies such as microarrays and next-generation sequencing (NGS), increasing in-depth studies of tumor biology were spurred at the genetic and genomic level, leading to better targeted and personalized healthcare solutions for cancer patients. The successful implementation of the human genome project has made people realize that genetic, environmental, and lifestyle factors should be combined together to study cancer due to its complexity. For example, some malignant tumors have been proven to be related to the mutations of a drive gene by using specific monoclonal antibodies and small molecule compounds to block or suppress the relevant molecular targets that can inhibit tumor growth and metastasis or induce apoptosis; the survival time of patients has been significantly extended.

The increasing availability and growth rate of “Big Data” derived from various omics open a new window to improve clinical diagnoses or therapeutics of cancer, but there are many challenges in efficient analysis and interpretation of such big and complex data. For instance, how to manage, extract, analyze, integrate, visualize, and communicate the hidden information from the myriad of data representations of cancer evolved into one of the greatest challenges in next-generation biomedicine. Thus, there is a need to fundamentally address all the above-mentioned issues in Big Data in cancer healthcare.

There are six interesting research papers in this special issue covering machine learning methods on feature selection of gene expression profile, cancer prediction, potential new drug design, and QSAR study of anticancer drugs.

Gene expression profiles provide a new insight into cancer diagnosis at a molecular level which paved the way towards personalized medicine. Gene expression data usually contains a large number of genes, but a small number of samples. Feature selection for gene expression data aims at finding a set of genes that best discriminate biological samples of different types. Q. Su et al. proposed a gene subset selection algorithm based on the Kolmogorov-Smirnov (K-S) test and correlation-based feature selection (CFS) principles to address the challenging problem of selecting distinguished genes from cancer gene expression datasets. The authors compared the K-S test plus CSF with K-S test alone, CFS alone, ReliefF, and mRMR feature selection in 5 cancer gene expression datasets, which adopted support vector machines (SVM) as the classification tool and used the criteria of accuracy to evaluate the performance of the classifiers on the selected gene subsets. The results show that this combination algorithm is more efficient.

L. Yang et al. presented a gene subset selection algorithm RS_SVM based on aggregating SVMs trained on eight random subspaces gene expression profiles. The results show that RS_SVM outperforms single SVM, KNN, CART, Bagging, AdaBoost, and 16 state-of-the-art methods in literatures. L. Yang et al.'s study provides a potential tool for the problems of high dimension and small sample problem in gene expression data which could lead to overfitting and huge computing pressure. The authors also proposed that RS_SVM is not suitable for heterogeneous data as they failed to apply RS_SVM with PCA on two gene expression profiles.

Glioma is the most common and most aggressive malignant brain tumor in humans that affects nonneural glial cells in the central nervous system. The knowledge of glioblastoma at the molecular and structural level will greatly improve the treatment of glioma in the clinic. H. Long et al. made a PPI network of key DEGs to study the significant functions associated with the occurrence and development of glioma combined with enriched GO and KEGG data. Pathways in cancer, MAPK signaling pathway, focal adhesion, and calcium signaling pathway were regarded to be related to the occurrence of glioma. In addition, some key genes such as MMP9, CD44, CDC42, COL1A1, COL1A2, CAMK2A, and CAMK2B were also proposed, which might be target genes for diagnosing glioblastoma.

EGFR is considered to be an anticancer target as it has been found in some solid tumors, such as glioma, lung cancer, ovarian cancer, breast cancer, and other cancers. Several efforts have been made to develop EGFR inhibitors for the treatment of cancer. The low selectivity, high toxicity, and reduced activity promote the design of improved EGFR. M. Zhao et al. introduced the application of 2D and 3D QSAR methods to discriminate EGFR inhibitors and subsequently performed structural docking of the molecules. Overall, this study is modest but nice which contributes to a deeper understanding of the intricacies of drug potency for inhibiting EGFR.

J. Chen et al. developed a novel monocarbonyl curcumin analog which exhibits preferable anticancer effects on laryngeal cancer cells via targeting NF-*κ*B with little toxicity to normal cells. Many traditional Chinese medicine extracts have preferable anticancer effects; however, their toxicities are usually neglected. Meanwhile, this study also reveals that NF-*κ*B is probably a potential target for laryngeal cancer treatment using molecular docking method. Therapeutics based on targeting NF-*κ*B may be effective approaches for laryngeal cancer treatment in the future. However, the results of this study all come from in vitro trials; further tests of this curcumin analog in vivo need to be performed.

M.C. Ng et al. built a bioactivity model for complex mixtures of herb Radix Astragali (RA) extracts based on chemical fingerprinting profiles with Elastic Net Partial Least Square (EN-PLS) algorithm. The prediction platform they obtained has the capacity to identify potential key bioactivity-related chemical components of the herb, which is helpful for discovering potential novel drugs, especially for the herbal extracts to be used in clinical trials.

*Bing Niu* *Bing Niu* *Peter B. Harrington* *Peter B. Harrington* *Guozheng Li* *Guozheng Li* *Jianxin Li* *Jianxin Li* *Simon Poon* *Simon Poon*

